# Neoadjuvant Chemohormonal Therapy in Prostate Cancer Before Radical Prostatectomy: A Systematic Review and Meta-Analysis

**DOI:** 10.3389/fonc.2022.906370

**Published:** 2022-05-11

**Authors:** Qingyu Ge, Hewei Xu, Dezhou Yue, Zongyao Fan, Zhengsen Chen, Jie Xu, Yiduo Zhou, Sicong Zhang, Jun Xue, Baixin Shen, Zhongqing Wei

**Affiliations:** ^1^Department of Urology, The Second Affiliated Hospital of Nanjing Medical University, Nanjing, China; ^2^Department of Urology, The Second Clinical Medical College of Nanjing Medical University, Nanjing, China

**Keywords:** prostate cancer, neoadjuvant chemohormonal therapy, radical prostatectomy, survival, meta-analysis

## Abstract

**Objective:**

This meta-analysis was to investigate the effects of neoadjuvant chemohormonal therapy (NCHT) on patients with prostate cancer (PCa) before radical prostatectomy (RP) and attempt to provide meaningful evidence.

**Methods:**

A systematic search was performed using the PubMed, Web of Science, and Cochrane Library databases in February 2022 based on the Preferred Reporting Items for Systematic Reviews and Meta-Analyses guidelines. The relevant studies were critically screened and we extracted the data of demography, postoperative pathology, and survival to calculate the pooled effect sizes. Subgroup analyses and sensitivity analyses were used to explore the source of heterogeneity.

**Results:**

Six identified studies involving 1717 subjects were included according to the selection criteria. There was no significant difference between NCHT plus RP and RP alone groups regarding lymph node involvement (risk ratio [RR]=1.03, 95% confidence interval [CI]: 0.57-1.87, P=0.92). However, NCHT prior to RP significantly decreased the rates of positive surgical margin (PSM, RR=0.35, 95% CI: 0.22-0.55, P<0.0001) and seminal vesicle invasion (SVI, RR=0.78, 95% CI: 0.65-0.95, P=0.01), and increase pathological downstaging (RR=1.64, 95% CI: 1.17-2.29, P=0.004). Additionally, biochemical recurrence-free survival (BRFS) and overall survival (OS) were significantly prolonged under the administration of NCHT (HR=0.54, 95% CI: 0.34-0.85, P=0.008 and HR=0.67, 95% CI: 0.48-0.94, P=0.02, respectively).

**Conclusions:**

Compared to the RP alone group, patients with NCHT plus RP showed significant improvements in PSM, SVI, pathological downstaging, BRFS, and OS, whereas further multicenter randomized controlled trials are needed to consolidate this concept.

## Introduction

Prostate cancer (PCa) is one of the most common genitourinary malignancies and the second leading cause of cancer mortality in men worldwide (1), and approximately 15% present with high-risk localized disease in newly diagnosed PCa (2). To date, no consensus is available on the optimal treatment strategies for men with high-risk localized PCa (3) and these patients suffer an increased risk of biochemical recurrence and cancer-related death following radical prostatectomy (RP). The multimodal therapy strategy including systemic and local therapies has been raised for years and the majority of urologists are inclined to perform radical prostatectomy plus extended pelvic lymph node dissection or external beam radiotherapy plus androgen deprivation therapy (ADT) (4).

Administration of neoadjuvant hormone therapy (NHT) before RP can reduce positive surgical margin (PSM) rate, prostate volume, and pathologic stage, whereas survival benefits such as biochemical recurrence-free survival (BRFS) or overall survival (OS) are not observed (5). Although numerous prospective and retrospective studies aim to find a better prognosis associated with NHT, the European Association of Urology notes that evidence of neoadjuvant ADT is weak and has limited recommendations for initiating NHT before surgery. Hence, for improving oncological outcomes, chemotherapy, which is widely used in leukemia and other solid tumors (6, 7), has been administrated in men with metastatic PCa and shown to prolong survival in advanced PCa (8, 9). In addition, some clinical trials have preliminarily revealed that neoadjuvant chemohormonal therapy (NCHT) was well tolerated and showed acceptable therapeutic effects (10–12).

Based on these findings, a growing number of investigators were committed to exploring the significant differences between NCHT and NHT or RP alone recently, especially in survival analysis. However, because of the lack of high-level evidence, namely multicenter randomized controlled trials (RCTs) and meta-analyses, the present guidelines make strong recommendations difficult. In this meta-analysis, we aimed to evaluate the perioperative and survival outcomes of NCHT prior to RP, and provide the available evidence for supporting the potential advantages of NCHT.

## Materials and Methods

### Search Strategy

This meta‐analysis was conducted based on the Preferred Reporting Items for Systematic Reviews and Meta-Analyses (PRISMA) (13), and the review protocol was registered on PROSPERO (CRD42022321236). We searched relevant studies from PubMed, Web of Science, and Cochrane Library databases in February 2022, and the language of publications was restricted to English only. The medical subject heading (MeSH) terms were used as follows: (“Prostatic Neoplasms”[MeSH]) AND (“Prostatectomy”[MeSH]) AND (“Neoadjuvant Therapy”[MeSH]), and the detailed strategies were available in **Supplementary Material**. The relevant cited references from the selected studies were also retrieved to ascertain potentially acceptable literature.

### Study Selection

All randomized and non-randomized studies that met the inclusion criteria were included (1): the authors compared NCHT plus RP with RP alone (2); the study reported at least one of the pathological and survival outcomes and sufficient data for this analysis (3); the study was a cohort study with full text rather than reviews, meta‐analyses, case reports, meeting abstracts, editorials, and general commentaries. Two reviewers independently selected the eligible studies according to the inclusion criteria and controversies were resolved by a third reviewer. In the presence of duplicate publications, we included the higher quality.

### Data Extraction and Quality Assessment

Two authors respectively extracted information from included papers as follows: first author, publication year, country, study design, clinical intervention, number of subjects, age (mean or median), prostate-specific antigen (PSA, mean or median), follow-up duration (median), perioperative outcomes (PSM, pathological downstaging (14), lymph node involvement [LNI], and seminal vesicle invasion [SVI]), and survival outcomes (BRFS, OS). Disagreements were settled by discussing with a third investigator.

The quality of the RCTs was evaluated using the Cochrane risk-of-bias tool (15), and the Newcastle-Ottawa Scale (NOS) was adopted for assessing the cohort studies (16). The categories of NOS include Selection, Comparability, and Outcome (four, two, and three stars maximally, respectively), and studies that graded more than six stars were regarded as high quality.

### Statistical Analysis

The survival outcomes were pooled as hazard ratios (HRs) with 95% confidence intervals (CIs), and the perioperative outcomes were pooled as risk ratios (RRs) with 95% CIs. We used the Cochrane Q test and *I*^2^ statistics to assess the heterogeneity, and a random-effect model was employed when P value<0.05 or *I*^2^>50% (i.e. significant heterogeneity), or else a fix-effect model was adopted. Additionally, the sources of heterogeneity were investigated through sensitivity analyses and subgroup analyses. The publication bias could be preliminarily identified *via* funnel plots and quantified based on Egger’s test. All data analyses were performed by Review Manager Version 5.4 (The Cochrane Collaboration, Oxford, UK) and Stata Version 16.0 software (Stata Corp., College Station, TX, USA), and P <0.05 was considered statistically significant.

## Results

### Description of Eligible Studies

**Figure 1:**
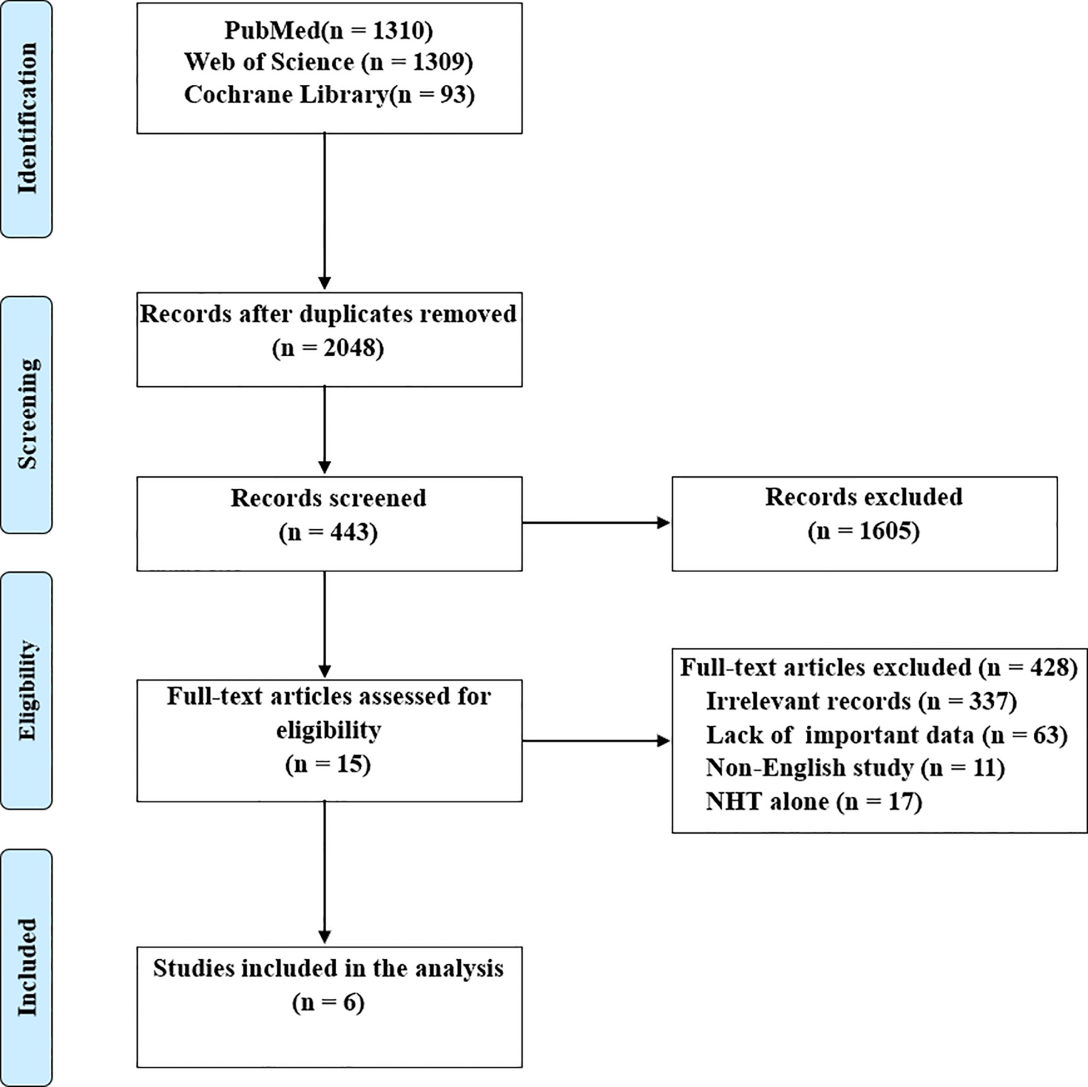
Preferred Reporting Items for Systematic Reviews and Meta-analysis (PRISMA) flow diagram.

The details of literature retrieval and screening are shown in **Figure 1**. A total of 2712 studies were initially searched from the above-mentioned three databases. Under the inclusion criteria, six articles with 1717 subjects (17–22), ranging from 2016 to 2021, were involved in this meta‐analysis following screening the titles, abstracts, and full-text.

As shown in **Table 1**, two publications were RCTs (19, 21) and four papers were non-randomized prospective (22) or retrospective cohort studies (17, 18, 20), two of which were also propensity score match analyses (19, 22). There were 998 and 719 patients who received NCHT plus RP and RP alone, respectively, and the majority of studies administrated docetaxel to patients (17, 20–22), otherwise estramustine phosphate (18) alone, or docetaxel plus estramustine (19). The median or mean age ranged from 62 to 69 years and the median or mean PSA (ng/mL) ranged from 9.5 to 97.7. Four studies reported the follow-up durations (17, 18, 20, 21), differing from 12.5 months to 141.6 months.

**Table 1:** Baseline characteristics and interventions included in the meta-analysis.

Author	Year	Country	Study design	Interventions	Sample size		Age (years)		PSA level (ng/ml)		Follow−up(months)	
					NCHT+RP	RP	NCHT+RP	RP	NCHT+RP	RP	NCHT+RP	RP
Nosov et al. (17)	2016	Russia	Retrospective	Docetaxel + prednisolone + RP versus RP alone	21	23	64^a^	65^a^	28.5^a^	31.9^a^	141.6^a^	128.4^a^
Fujita et al. (18)	2017	Japan	Retrospective	Estramustine phosphate + GnRH agonist or antagonist versus RP alone	436	177	68^a^	68^a^	9.94^a^	11.8^a^	48.8^a^	111^a^
Narita et al. (19)	2019	Japan	RCT	Docetaxel + estramustine + androgen blockade + RP versus RP alone	56	56	65.4^b^	66.4^b^	26.7^b^	23.4^b^	NA	NA
Pan et al. (20)	2019	China	Retrospective	Docetaxel +androgen blockade + RP versus RP alone	60	44	65^a^	69^a^	93.2^a^	60.3^a^	12.5^a^	22.8^a^
Eastham et al. (21)	2020	Multicenter	RCT	Docetaxel + androgen deprivation +RP versus RP alone	391	397	62^a^	63^a^	9.5^a^	10.2^a^	73.2^a^	
Chi et al. (21)	2021	China	Prospective	Docetaxel + leuprorelin/goserelin +prednisone + RP versus RP alone	34	22	66.0^b^	68.29^b^	97.7^b^	75^b^	NA	NA

^a^Median, ^b^Mean.

PSA, prostate-specific antigen; NCHT, neoadjuvant chemohormonal therapy; RP, radical prostatectomy; RCT, randomized controlled trial.

### Quality Evaluation

We assessed the risk of bias for two RCTs (**Supplementary Figure 1**) and calculated the score for four cohort studies (**Table S1**) as per the respective guideline. In the study by Narita et al. (19), random sequence generation was not mentioned and Eastham et al. (21) did not mention the reasons for dropouts. Moreover, both RCTs did not describe how outcomes were assessed blind. For the four non-randomized studies, the scores ranged from 6 to 9 stars, signifying that all included studies were eligible for subsequent meta-analysis.

### Meta-Analysis

#### Pathological Outcomes

**Figure 2:**
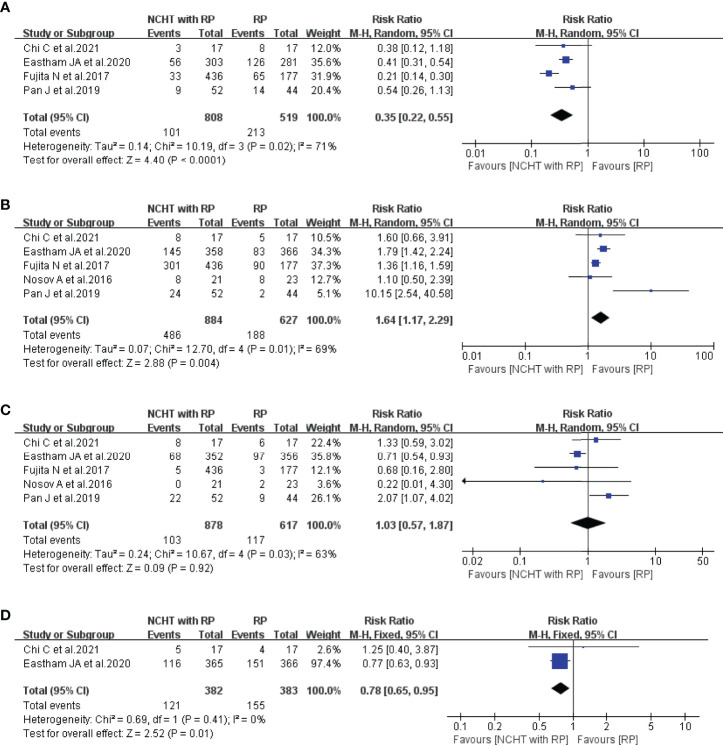
Meta-analyses of the pathological outcomes of patients administered NCHT plus RP versus RP alone (risk ratios). **(A)** positive surgical margin, **(B)** pathological downstaging, **(C)** lymph node involvement, **(D)** seminal vesicle invasion. NCHT, neoadjuvant chemohormonal therapy; RP, radical prostatectomy; CI, confidence interval.

We firstly analyzed the differences of PSM, pathological downstaging, LNI, and SVI between NCHT plus RP and RP alone. As illustrated in **Figure 2**, NCHT before RP could significantly reduce the proportion of PSM (RR=0.35, 95% CI: 0.22-0.55, P<0,0001, *I*^2^ = 71%, **Figure 2A**) and SVI (RR=0.78, 95% CI: 0.65-0.95, P=0.01, *I*^2^ = 0%, **Figure 2D**), and increase the rate of pathological downstaging (RR=1.64, 95% CI: 1.17-2.29, P=0.004, *I*^2^ = 69%, **Figure 2B**). However, no significant difference was observed in LNI (RR=1.03, 95% CI: 0.57-1.87, P=0.92, *I*^2^ = 63%, **Figure 2C**) with or without NCHT.

#### Survival Outcomes

**Figure 3:**
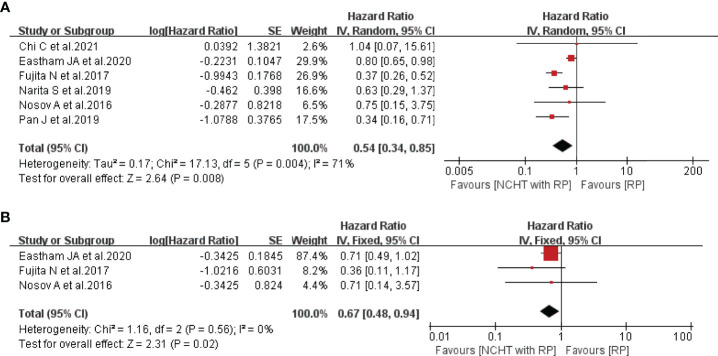
Forest plot of hazard ratios for survival outcomes with NCHT plus RP compared with RP alone. **(A)** biochemical recurrence-free survival, **(B)** overall survival. NCHT, neoadjuvant chemohormonal therapy; RP, radical prostatectomy; CI, confidence interval, SE, standard error.

All included studies provided the data of BRFS and the patients receiving NCHT characterized with significantly prolonged BRFS (HR=0.54, 95% CI: 0.34-0.85, P=0.008, *I*^2 ^= 71%, **Figure 3A**). Furthermore, Fujita et al. (18) and Eastham et al. (21) reported that NCHT was related to improved OS, thus the meta-analysis demonstrated that NCHT does significantly ameliorate the OS without any heterogeneity (HR=0.67, 95% CI: 0.48-0.94, P=0.02, *I*^2^ = 0%, **Figure 3B**).

### Subgroup Analyses and Sensitivity Analyses

In consideration of the heterogeneity, subgroup analyses (**Table 2**) and sensitivity analyses (**Supplementary Figure 2**) were performed to assess the stability of the above results. The subgroup analyses indicated that there was no significant difference in the PSM and pathological downstaging between NCHT plus RP and RP alone. However, the sample size plus region and the study design combined with the region were the main sources of high heterogeneity in LNI and BRFS, respectively.

**Table 2:** Subgroup analyses of selective outcome indicators according to sample size, region, and study design.

Characteristic	Number of studies	*I*^2^ (%)	P_heterogeneity_	RR/HR (95% CI)	P value^a^	P value^b^
**PSM**						
Overall	4	71	0.02	0.35 (0.22-0.55)	<0.01	
Region						0.52
Asia	3	65	0.06	0.32 (0.16-0.64)	<0.01	
Non-Asia	1	–	–	0.41 (0.31-0.54)	<0.01	
Sample size						0.28
≥200	2	88	<0.01	0.30(0.15-0.58)	<0.01	
<200	2	0	0.59	0.49 (0.26-0.91)	0.02	
Study design						0.61
Prospective	2	0	0.87	0.41 (0.32-0.53)	<0.01	
Retrospective	2	81	0.02	0.32 (0.12-0.82)	0.02	
**Pathological Downstaging**						
Overall	5	69	0.01	1.64 (1.17-2.29)	<0.01	
Region						0.52
Asia	3	78	0.01	2.28 (0.88-5.94)	0.09	
Non-Asia	2	28	0.24	1.62 (1.11-2.37)	0.01	
Sample size						0.52
≥200	2	75	0.05	1.54 (1.17-2.02)	<0.01	
<200	3	79	<0.01	2.34 (0.67-8.11)	0.18	
Study design						0.84
Prospective	2	0	0.81	1.77 (1.42-2.21)	<0.01	
Retrospective	3	78	0.01	1.95 (0.80-4.73)	0.14	
**LNI**						
Overall	5	63	0.03	1.03 (0.57-1.87)	0.92	
Region						<0.01
Asia	3	8	0.34	1.54(0.92-2.56)	0.10	
Non-Asia	2	0	0.44	0.70(0.54-0.92)	0.01	
Sample size						0.02
≥200	2	0	0.95	0.71 (0.54-0.93)	0.01	
<200	3	20	0.29	1.57 (0.85-2.91)	0.15	
Study design						0.69
Prospective	2	52	0.15	0.86 (0.49-1.53)	0.61	
Retrospective	3	47	0.15	1.11 (0.36-3.37)	0.86	
**BRFS**						
Overall	6	71	<0.01	0.54 (0.34-0.85)	<0.01	
Region						<0.01
Asia	4	0	0.54	0.40 (0.30-0.53)	<0.01	
Non-Asia	2	0	0.94	0.80 (0.65-0.98)	0.03	
Sample size						0.80
≥200	2	93	<0.01	0.55 (0.26-1.17)	0.12	
<200	4	0	0.59	0.49 (0.30-0.81)	<0.01	
Study design						<0.01
Prospective	3	0	0.83	0.79 (0.65-0.96)	0.02	
Retrospective	3	0	0.68	0.37 (0.27-0.51)	<0.01	

^a^ Test for overall effect, ^b^ Test for subgroup differences.

PSM, positive surgical margin; LNI, lymph node involvement; BRFS, biochemical recurrence-free survival; RR, risk ratio; HR, hazard ratio.

Next, sensitivity analyses were conducted by omitting one study in turn. As shown in **Supplementary Figure 2**, the pooled RRs for PSM, pathological downstaging, and LNI, and the pooled HRs for BRFS were not significantly varied, stating the consistency of our study.

### Publication Bias

Finally, we applied funnel plot analysis and Egger’s test to explore the possible publication bias in this study. The funnel plots for PSM, pathological downstaging, LNI, SVI, BRFS, and OS were visually symmetrical (**Supplementary Figure 3**), which were consistent with the results of Egger’s test (P=0.672, P=0.169, P=0.470, P=0.408, P=0.773, and P=0.496, respectively), manifesting no significant publication bias.

## Discussion

The definition of high-risk localized PCa is PSA level>20 ng/mL or Gleason score≥8, scilicet International Society of Urological Pathology (ISUP) grade 4–5 or clinical stage≥cT2c (3, 23). Currently, there is still no consensus on optimum therapeutic strategies for high-risk PCa, not to mention very high-risk PCa, which was proposed by Sundi et al. (24) and characterized with higher metastasis risk and cancer-specific mortality. In addition to the recommended options in predominant international guidelines, since docetaxel was found to be significantly superior in terms of survival benefits in metastatic castration-resistant prostate cancer (25), chemotherapy, especially docetaxel, attracted extensive attention. The CHAARTED and STAMPEDE trial demonstrated the efficacy of docetaxel for metastatic hormone-sensitive prostate cancer with acceptable adverse events in 2015 and 2016 (8, 9), respectively. Furthermore, a meta-analysis showed that additional chemotherapy to ADT could significantly improve progression-free survival in high-risk PCa (26). These findings made it logical to examine chemotherapy earlier in the process of PCa and we believed that NCHT could be considered as a part of multi-modal therapy based on this study.

Previous papers elucidated that decreased prostate volume after NHT might prompt surgeons to dissect smoother (27, 28), thereby resulting in lower operative difficulty theoretically, whereas controversy still exists regarding the advantages of NHT in optimizing surgical variables, including operation time and hemorrhage. In NCHT, despite the analysis of the two mentioned variables was absent in this study because of limited data, Nosov et al. (17) and Pan et al. (20) found no significant difference in operation time and blood loss between NCHT and RP alone group, namely NCHT did not increase the surgical difficulty. Of note, PSM plays a crucial role in predicting biochemical recurrence (29) and NCHT could improve the pathological outcomes of high-risk PCa in our study, such as reducing PSM and increasing the rate of pathological downstaging, which was consistent with a latest meta-analysis for NHT (30).

In general, patients and urologists may be more concerned with life expectancy. Administration of NHT before RP was evaluated in different RCTs showing no satisfactory effect on survival (14, 31, 32), which was also acknowledged in a systematic review (33). With regard to the reasons, neoadjuvant ADT cannot block the production of adrenal and intratumoral androgens to stop the sustainability of androgen receptor signalling (34). Moreover, the duration of follow-up and NHT was insufficient, and numerous subjects were enrolled in the early to middle stages of the disease, weakening the effects of NHT. On the contrary, the prevalent pharmaceuticals used in NCHT can restrain cancer cell proliferation and induce apoptosis by targeting microtubules or DNA (35). Patients receiving neoadjuvant docetaxel and/or estramustine phosphate demonstrated a significant improvement in BRFS and OS as expected (P=0.008 and P=0.02, respectively), which was the firm superiority compared to NHT.

Due to the lack of records of adverse events in the RP alone group (not placebo), we cannot conduct a meta-analysis about safety. Nonetheless, there was no significant difference in early and late postoperative complications between the NCHT plus RP and RP groups (17). The current included literature also revealed that the most common adverse events were neutropenia, fatigue, hot flashes, and other gastroenterological reactions (20–22). No chemotherapy-related deaths were observed and the rates of grade 3 and 4 adverse events were comparable to the patients undergoing ADT plus radiotherapy (36). Therefore, it is reasonable to assume that chemotherapy before RP is well tolerated (35).

Though the eligible studies possess a low risk of bias and most findings are positive, the heterogeneity is definitely present and some factors may be involved. First, the different definitions of high-risk PCa were utilized and Pan et al. even performed interventions on patients with very high-risk PCa (20). Second, the included studies implemented follow−up schedules in a different manner, and BRFS was defined as a serum PSA level >0.2 ng/mL with a diverse time interval. Additionally, Grasso et al. considered that the capacity to find significant differences was associated with methodology immensely, and study design and sample size are two of the most important elements (37), which may explain why sample size, region, and study design were the main sources of high heterogeneity in LNI and BRFS. However, no study was responsible for the heterogeneity under the sensitivity analyses.

Unfortunately, our work is limited by a small number of studies, especially published multicenter, prospective trials, hence our results may be affected by future papers. On the other hand, the application of the random-effect model was to reduce but cannot eliminate the influence of heterogeneity. Besides, other survival outcomes (cancer-specific survival and metastasis-free survival) are not analyzed, which may provide more clinically meaningful information.

## Conclusions

The results of our meta-analysis suggest that NCHT may improve pathological outcomes, manifesting as depressed PSM and SVI rates, and elevated pathological downstaging rate. More importantly, prolonged BRFS and OS are available following NCHT plus RP and further large-scale RCTs are necessary to offer more powerful evidence.

## Data Availability Statement

The original contributions presented in the study are included in the article/**Supplementary Material**. Further inquiries can be directed to the corresponding author.

## Author Contributions

QG, HX, and ZW contributed to conception and design of the study. DY, ZF, ZC, JiX, and YZ were in charge of data collection. SZ, JuX, and BS performed the statistical analysis. QG wrote the first draft of the manuscript and HX plus ZW edited the manuscript. All authors contributed to manuscript revision, read, and approved the submitted version.

## Funding

This study was funded by the National Key Technology R&D Program of China (nos. 2018YFC2002204) and the Postgraduate Research & Practice Innovation Program of Jiangsu Province (JX11213894).

## Conflict of Interest

The authors declare that the research was conducted in the absence of any commercial or financial relationships that could be construed as a potential conflict of interest.

## Publisher’s Note

All claims expressed in this article are solely those of the authors and do not necessarily represent those of their affiliated organizations, or those of the publisher, the editors and the reviewers. Any product that may be evaluated in this article, or claim that may be made by its manufacturer, is not guaranteed or endorsed by the publisher.

## Supplementary Material

Supplementary Figure 1 Risk of bias and risk of bias summary for included randomized controlled trials.

Supplementary Figure 2 Sensitivity analysis of pooled positive surgical margin **(A)**, pathological downstaging **(B)**, lymph node involvement **(C)**, and biochemical recurrence-free survival **(D)**.

Supplementary Figure 3 Funnel plot of the studies for positive surgical margin **(A)**, pathological downstaging **(B)**, lymph node involvement **(C)**, seminal vesicle invasion **(D)**, biochemical recurrence-free survival **(E)**, and overall survival **(F)**.
