# Supplementary figures and images for: Neoadjuvant Chemohormonal Therapy in Prostate Cancer Before Radical Prostatectomy: A Systematic Review and Meta-Analysis
Source: Front Oncol. 2022 May 11;12:906370. doi: 10.3389/fonc.2022.906370 (PMC9130750; doi:10.3389/fonc.2022.906370)

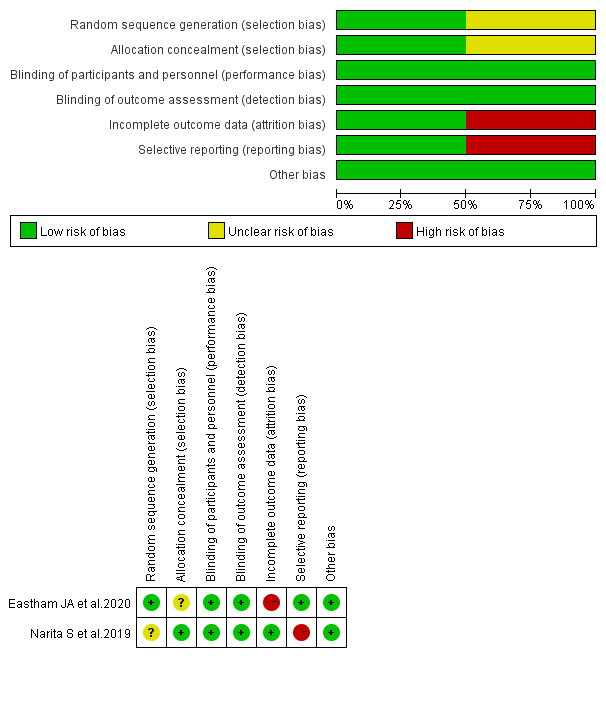

Supplement: Supplementary Figure 1 — Risk of bias and risk of bias summary for included randomized controlled trials. [file Image_1.tif]

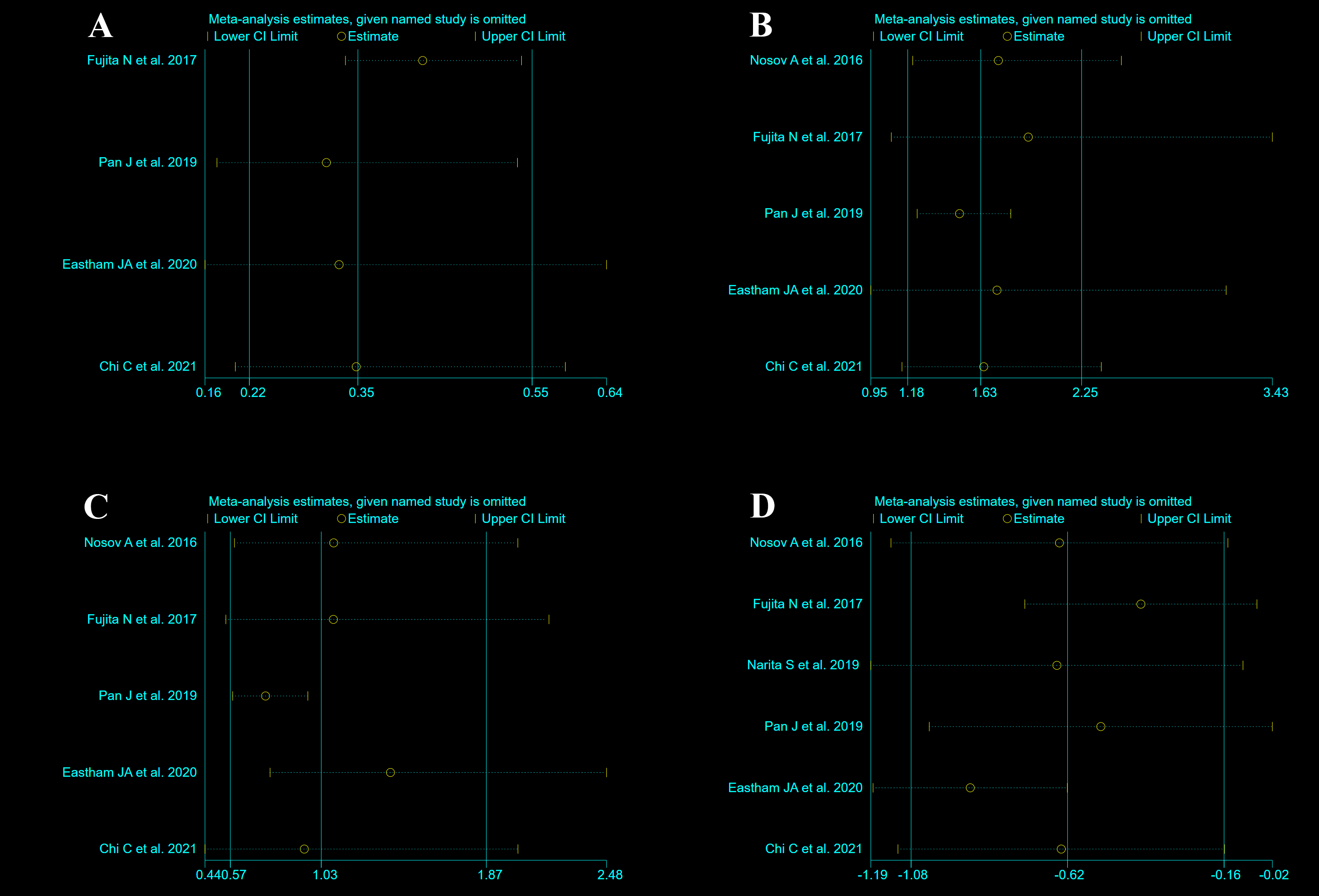

Supplement: Supplementary Figure 2 — Sensitivity analysis of pooled positive surgical margin (A), pathological downstaging (B), lymph node involvement (C), and biochemical recurrence-free survival (D). [file Image_2.tif]

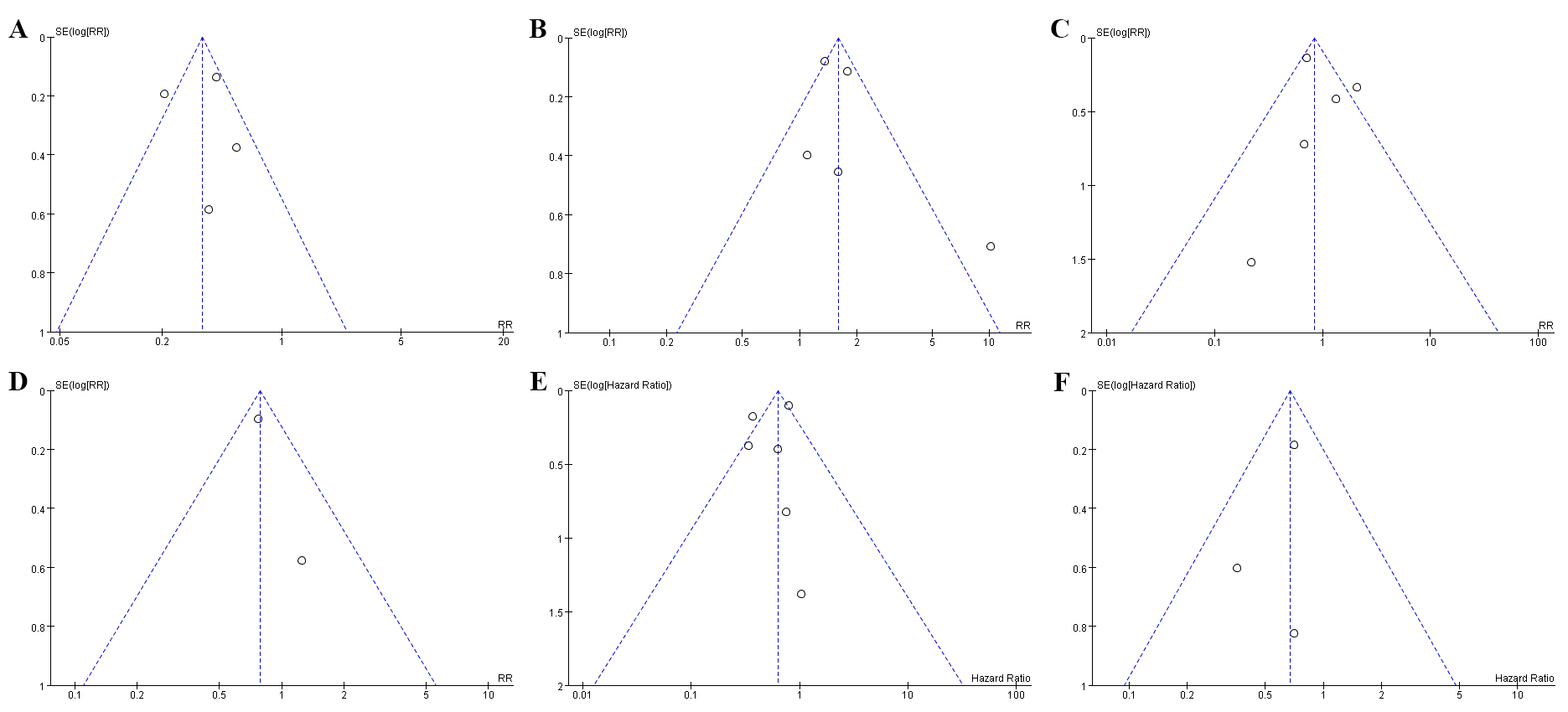

Supplement: Supplementary Figure 3 — Funnel plot of the studies for positive surgical margin (A), pathological downstaging (B), lymph node involvement (C), seminal vesicle invasion (D), biochemical recurrence-free survival (E), and overall survival (F). [file Image_3.tif]
