# Supplementary material for: Neoadjuvant Chemohormonal Therapy in Prostate Cancer Before Radical Prostatectomy: A Systematic Review and Meta-Analysis
Source: Front Oncol. 2022 May 11;12:906370. doi: 10.3389/fonc.2022.906370 (PMC9130750; doi:10.3389/fonc.2022.906370)
Supplement: Supplementary file 4 [file DataSheet_1.docx]

**Pubmed (n=1310)**

((((((((((((((((((("Prostatic Neoplasms"[Mesh]) OR (Prostate Neoplasms)) OR (Neoplasms, Prostate)) OR (Neoplasm, Prostate)) OR (Prostate Neoplasm)) OR (Neoplasms, Prostatic)) OR (Neoplasm, Prostatic)) OR (Prostatic Neoplasm)) OR (Prostate Cancer)) OR (Cancer, Prostate)) OR (Cancers, Prostate)) OR (Prostate Cancers)) OR (Cancer of the Prostate)) OR (Prostatic Cancer)) OR (Cancer, Prostatic)) OR (Cancers, Prostatic)) OR (Prostatic Cancers)) OR (Cancer of Prostate)) AND (((((((((("Prostatectomy"[Mesh]) OR (Prostatectomies)) OR (Prostatectomy, Suprapubic)) OR (Prostatectomies, Suprapubic)) OR (Suprapubic Prostatectomies)) OR (Suprapubic Prostatectomy)) OR (Prostatectomy, Retropubic)) OR (Prostatectomies, Retropubic)) OR (Retropubic Prostatectomies)) OR (Retropubic Prostatectomy))) AND ((((((((((((((((((((((((((((((((((((((((((((((((("Neoadjuvant Therapy"[Mesh]) OR (Neoadjuvant Therapies)) OR (Therapy, Neoadjuvant)) OR (Neoadjuvant Treatment)) OR (Neoadjuvant Treatments)) OR (Treatment, Neoadjuvant)) OR (Neoadjuvant Radiotherapy)) OR (Neoadjuvant Radiotherapies)) OR (Radiotherapy, Neoadjuvant)) OR (Neoadjuvant Radiation Treatment)) OR (Neoadjuvant Radiation Treatments)) OR (Radiation Treatment, Neoadjuvant)) OR (Treatment, Neoadjuvant Radiation)) OR (Neoadjuvant Radiation Therapy)) OR (Neoadjuvant Radiation Therapy)) OR (Radiation Therapy, Neoadjuvant)) OR (Therapy, Neoadjuvant Radiation)) OR (Neoadjuvant Radiation)) OR (Neoadjuvant Radiations)) OR (Radiation, Neoadjuvant)) OR (Neoadjuvant Systemic Therapy)) OR (Neoadjuvant Systemic Therapies)) OR (Systemic Therapy, Neoadjuvant)) OR (Therapy, Neoadjuvant Systemic)) OR (Neoadjuvant Systemic Treatment)) OR (Neoadjuvant Systemic Treatments)) OR (Systemic Treatment, Neoadjuvant)) OR (Treatment, Neoadjuvant Systemic)) OR (Neoadjuvant Chemotherapy)) OR (Chemotherapy, Neoadjuvant)) OR (Neoadjuvant Chemotherapies)) OR (Neoadjuvant Chemotherapy Treatment)) OR (Chemotherapy Treatment, Neoadjuvant)) OR (Neoadjuvant Chemotherapy Treatments)) OR (Treatment, Neoadjuvant Chemotherapy)) OR (Neoadjuvant Chemoradiotherapy)) OR (Chemoradiotherapy, Neoadjuvant)) OR (Neoadjuvant Chemoradiotherapies)) OR (Neoadjuvant Chemoradiation Therapy)) OR (Chemoradiation Therapy, Neoadjuvant)) OR (Neoadjuvant Chemoradiation Therapies)) OR (Therapy, Neoadjuvant Chemoradiation)) OR (Neoadjuvant Chemoradiation Treatment)) OR (Chemoradiation Treatment, Neoadjuvant)) OR (Neoadjuvant Chemoradiation Treatments)) OR (Treatment, Neoadjuvant Chemoradiation)) OR (Neoadjuvant Chemoradiation)) OR (Chemoradiation, Neoadjuvant)) OR (Neoadjuvant Chemoradiations))

**Web of Science (n=1309)**

#1: TS = (Prostatic Neoplasms OR Prostate Neoplasms OR Neoplasms, Prostate OR Neoplasm, Prostate OR Prostate Neoplasm OR Neoplasms, Prostatic OR Neoplasm, Prostatic OR Prostatic Neoplasm OR Prostate Cancer OR Cancer, Prostate OR Cancers, Prostate OR Prostate Cancers OR Cancer of the Prostate OR Prostatic Cancer OR Cancer, Prostatic OR Cancers, Prostatic OR Prostatic Cancers OR Cancer of Prostate)

#2: TS = (Prostatectomy OR Prostatectomies OR Prostatectomy, Suprapubic OR Prostatectomies, Suprapubic OR Suprapubic Prostatectomies OR Suprapubic Prostatectomy OR Prostatectomy, Retropubic OR Prostatectomies, Retropubic OR Retropubic Prostatectomies OR Retropubic Prostatectomy)

#3: TS = (Neoadjuvant Therapy OR Neoadjuvant Therapies OR Therapy, Neoadjuvant OR Neoadjuvant Treatment OR Neoadjuvant Treatments OR Treatment, Neoadjuvant OR Neoadjuvant Radiotherapy OR Neoadjuvant Radiotherapies OR Radiotherapy, Neoadjuvant OR Neoadjuvant Radiation Treatment OR Neoadjuvant Radiation Treatments OR Radiation Treatment, Neoadjuvant OR Treatment, Neoadjuvant Radiation OR Neoadjuvant Radiation Therapy OR Neoadjuvant Radiation Therapies OR Radiation Therapy, Neoadjuvant OR Therapy, Neoadjuvant Radiation OR Neoadjuvant Radiation OR Neoadjuvant Radiations OR Radiation, Neoadjuvant OR Neoadjuvant Systemic Therapy OR Neoadjuvant Systemic Therapies OR Systemic Therapy, Neoadjuvant OR Therapy, Neoadjuvant Systemic OR Neoadjuvant Systemic Treatment OR Neoadjuvant Systemic Treatments OR Systemic Treatment, Neoadjuvant OR Treatment, Neoadjuvant Systemic OR Neoadjuvant Chemotherapy OR Chemotherapy, Neoadjuvant OR Neoadjuvant Chemotherapies OR Neoadjuvant Chemotherapy Treatment OR Chemotherapy Treatment, Neoadjuvant OR Neoadjuvant Chemotherapy Treatments OR Treatment, Neoadjuvant Chemotherapy OR Neoadjuvant Chemoradiotherapy OR Chemoradiotherapy, Neoadjuvant OR Neoadjuvant Chemoradiotherapies OR Neoadjuvant Chemoradiation Therapy OR Chemoradiation Therapy, Neoadjuvant OR Neoadjuvant Chemoradiation Therapies OR Therapy, Neoadjuvant Chemoradiation OR Therapy, Neoadjuvant Chemoradiation OR Chemoradiation Treatment, Neoadjuvant OR Chemoradiation Treatment, Neoadjuvant OR Chemoradiation Treatment, Neoadjuvant OR Neoadjuvant Chemoradiation OR Chemoradiation, Neoadjuvant OR Neoadjuvant Chemoradiations)

#4: #1 AND #2 AND #3

**Cochrane Library (n=93)**

#1: [mh "Prostatic Neoplasms"]

#2: Prostate Neoplasms* OR Neoplasms, Prostate* OR Neoplasm, Prostate* OR Prostate Neoplasm* OR Neoplasms, Prostatic* OR Neoplasm, Prostatic* OR Prostatic Neoplasm* OR Prostate Cancer* OR Cancer, Prostate* OR Cancers, Prostate* OR Prostate Cancers* OR Cancer of the Prostate* OR Prostatic Cancer* OR Cancer, Prostatic* OR Cancers, Prostatic* OR Prostatic Cancers* OR Cancer of Prostate*: ti, ab, kw

#3: #1 OR #2

#4: [mh “Prostatectomy”]

#5: Prostatectomies* OR Prostatectomy, Suprapubic* OR Prostatectomies, Suprapubic* OR Suprapubic Prostatectomies* OR Suprapubic Prostatectomy* OR Prostatectomy, Retropubic* OR Prostatectomies, Retropubic* OR Retropubic Prostatectomies* OR Retropubic Prostatectomy*: ti, ab, kw

#6: #4 OR #5

#7: [mh “Neoadjuvant Therapy”]

#8: Neoadjuvant Therapies* OR Therapy, Neoadjuvant* OR Neoadjuvant Treatment* OR Neoadjuvant Treatments* OR Treatment, Neoadjuvant* OR Neoadjuvant Radiotherapy* OR Neoadjuvant Radiotherapies* OR Radiotherapy, Neoadjuvant* OR Neoadjuvant Radiation Treatment* OR Neoadjuvant Radiation Treatments* OR Radiation Treatment, Neoadjuvant* OR Treatment, Neoadjuvant Radiation* OR Neoadjuvant Radiation Therapy* OR Neoadjuvant Radiation Therapies* OR Radiation Therapy, Neoadjuvant* OR Therapy, Neoadjuvant Radiation* OR Neoadjuvant Radiation* OR Neoadjuvant Radiations* OR Radiation, Neoadjuvant* OR Neoadjuvant Systemic Therapy* OR Neoadjuvant Systemic Therapies* OR Systemic Therapy, Neoadjuvant* OR Therapy, Neoadjuvant Systemic* OR Neoadjuvant Systemic Treatment* OR Neoadjuvant Systemic Treatments* OR Systemic Treatment, Neoadjuvant* OR Treatment, Neoadjuvant Systemic* OR Neoadjuvant Chemotherapy* OR Chemotherapy, Neoadjuvant* OR Neoadjuvant Chemotherapies* OR Neoadjuvant Chemotherapy Treatment* OR Chemotherapy Treatment, Neoadjuvant* OR Neoadjuvant Chemotherapy Treatments* OR Treatment, Neoadjuvant Chemotherapy* OR Neoadjuvant Chemoradiotherapy* OR Chemoradiotherapy, Neoadjuvant* OR Neoadjuvant Chemoradiotherapies* OR Neoadjuvant Chemoradiation Therapy* OR Chemoradiation Therapy, Neoadjuvant* OR Neoadjuvant Chemoradiation Therapies* OR Therapy, Neoadjuvant Chemoradiation* OR Therapy, Neoadjuvant Chemoradiation* OR Chemoradiation Treatment, Neoadjuvant* OR Chemoradiation Treatment, Neoadjuvant* OR Chemoradiation Treatment, Neoadjuvant* OR Neoadjuvant Chemoradiation* OR Chemoradiation, Neoadjuvant* OR Neoadjuvant Chemoradiations*: ti, ab, kw

#9: #7 OR #8

#10: #3 AND #6 AND #9
