# Supplementary material for: Neoadjuvant Chemohormonal Therapy in Prostate Cancer Before Radical Prostatectomy: A Systematic Review and Meta-Analysis
Source: Front Oncol. 2022 May 11;12:906370. doi: 10.3389/fonc.2022.906370 (PMC9130750; doi:10.3389/fonc.2022.906370)
Supplement: Supplementary file 5 [file Table_1.docx]

**Table S1** Quality assessment of the cohort studies

| Study | Representativeness of the exposed cohort | Selection of the non-exposed cohort | Ascertainment of exposure | Outcome of interest was not present at start of study | Comparability of cohorts on the basis of the design or analysis | Assessment of outcome | Follow-up long enough for outcomes to occur | Adequacy of follow-up of cohorts | Total |
| --- | --- | --- | --- | --- | --- | --- | --- | --- | --- |
| Nosov et al., 2016^15^ | **★** | **★** | **★** | **★** | **★★** | **★** | **★** | **★** | **9** |
| Fujita N et al., 2017^16^ | **★** | **★** | **★** | **★** | **-** | **★** | **★** | **★** | **7** |
| Pan J et al., 2019^18^ | **★** | **★** | **★** | **★** | **★** | **-** | **-** | **★** | **6** |
| Chi C et al., 2021^20^ | **★** | **★** | **★** | **★** | **★** | **★** | **-** | **★** | **7** |
